# Can brain network connectivity facilitate the clinical development of disease-modifying anti-Alzheimer drugs?

**DOI:** 10.1093/braincomms/fcae460

**Published:** 2024-12-18

**Authors:** Lorenzo Pini, Simone Lista, Alessandra Griffa, Gilles Allali, Bruno P Imbimbo

**Affiliations:** Department of Neuroscience, Università degli Studi di Padova, 35121 Padova, Italy; Padova Neuroscience Center, Università degli Studi di Padova, 35121 Padova, Italy; i+HeALTH Strategic Research Group, Department of Health Sciences, Miguel de Cervantes European University (UEMC), Valladolid 47012, Spain; Department of Clinical Neurosciences, Leenaards Memory Center, Lausanne University Hospital and University of Lausanne, Montpaisible 16, 1011 Lausanne, Switzerland; Medical Image Processing Laboratory, Neuro-X Institute, École Polytechnique Fédérale De Lausanne (EPFL), Campus Biotech Chemin des Mines 9, 1202 Geneva, Switzerland; Department of Clinical Neurosciences, Leenaards Memory Center, Lausanne University Hospital and University of Lausanne, Montpaisible 16, 1011 Lausanne, Switzerland; Department of Research and Development, Chiesi Farmaceutici, 43122 Parma, Italy

**Keywords:** neurodegeneration, functional MRI, disease-modifying drugs, anti-amyloid, anti-tau

## Abstract

The preclinical phase of Alzheimer’s disease represents a crucial time window for therapeutic intervention but requires the identification of clinically relevant biomarkers that are sensitive to the effects of disease-modifying drugs. Amyloid peptide and tau proteins, the main histological hallmarks of Alzheimer’s disease, have been widely used as biomarkers of anti-amyloid and anti-tau drugs. However, these biomarkers do not fully capture the multiple biological pathways of the brain. Indeed, robust amyloid-target engagement by anti-amyloid monoclonal antibodies has recently translated into modest cognitive and clinical benefits in Alzheimer’s disease patients, albeit with potentially life-threatening side effects. Moreover, targeting the tau pathway has yet to result in any positive clinical outcomes. Findings from computational neuroscience have demonstrated that brain regions do not work in isolation but are interconnected within complex network structures. Brain connectivity studies suggest that misfolded proteins can spread through these connections, leading to the hypothesis that Alzheimer’s disease is a pathology of network disconnectivity. Based on these assumptions, here we discuss how incorporating brain connectivity outcomes could better capture global brain functionality and, in conjunction with traditional Alzheimer’s disease biomarkers, could facilitate the clinical development of new disease-modifying anti-Alzheimer’s disease drugs.

## Teaser

Pin *et al*. propose a new paradigm in the field of pharmacological research for Alzheimer’s disease. According to this new perspective, brain connectivity could play a key role in the search for pharmacological targets, in patient selection for clinical trials, and finally, as an efficacy marker to evaluate pharmaceutical compounds.

## Graphical Abstract

**Graphical Abstract:**
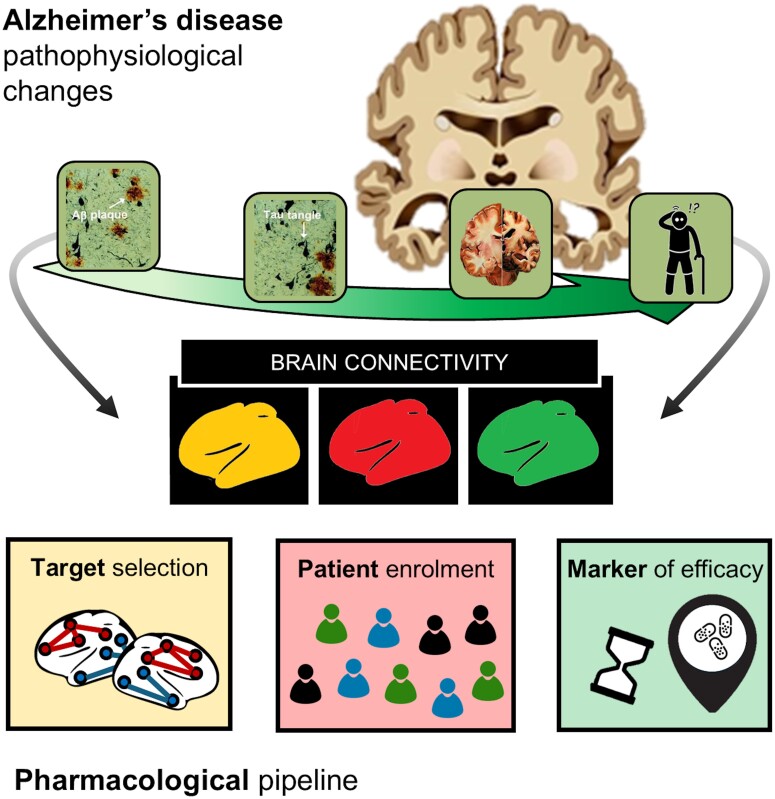

## Introduction

Over the last years, we have seen great progress in understanding the biological mechanisms underlying Alzheimer’s disease.^1,2^ Amyloid (Aβ) and tau levels from blood and CSF biomarkers are increasingly used to evaluate target engagement of anti-Aβ and anti-tau drugs. Recently, the Food and Drug Administration approved three anti-Aβ monoclonal antibodies (aducanumab, lecanemab and donanemab) for the treatment of early Alzheimer’s disease. While these drugs efficiently and rapidly clear brain Aβ plaques down to normal levels, they seem unable to reverse or halt clinical decline. Although commercialization for aducanumab has been discontinued mainly for adverse events (amyloid-related imaging abnormalities), lecanemab and donanemab significantly slowed cognitive decline. These effects were associated with marked and rapid decrease in brain Aβ load, although with frequent and sometimes life-threatening side effects.^3^ Patients with Alzheimer’s disease treated with lecanemab showed a significant increase in CSF Aβ42 (but not of Aβ40).^4^ Similarly, CSF levels of total-tau (t-tau), phosphorylated-tau at position 181 (p-tau181), and neurogranin were reduced by lecanemab treatment.^4^ A Phase 3 study investigating donanemab showed a marked decrease in Aβ-PET levels (not significant for Aβ42/Aβ40 plasma ratio and plasma p-tau217).^5^ After re-examining its initial opinion judging the clinical benefits (slowing of cognitive decline) not counterbalanced by the risk of serious side effects, the European Medicines Agency has recently approved the marketing authorization for lecanemab for a restricted indication in adults with early Alzheimer’s disease who have only 1 or no copy of apolipoprotein E4 (ApoE4). Similarly, tau protein plays a critical role in Alzheimer’s disease pathogenesis, as intra-neuronal deposits of tau correlate with clinical decline. Anti-tau antibodies can cross into the brain and enter neurons. However, to date several anti-tau monoclonal antibodies have failed to show clinical benefits in Alzheimer’s disease despite their ability to lower levels of p-tau. Semorinemab dose dependently increased plasma levels of mid-domain tau and lowered CSF p-tau181, p-tau217 and t-tau levels but did not improve cognitive scores in Alzheimer’s disease.^6^ Gosuranemab also showed target engagement by lowering CSF N-terminal tau levels but did not positively affect cognitive performance.^7^ Tilavonemab reduced CSF free tau levels in a dose-dependent manner and increased plasma t-tau, also indicating target engagement, but did not improve cognition in early Alzheimer’s disease.^8^ In a Phase 2 study in patients with mild Alzheimer’s disease, MAPT_rx_, an anti-tau antisense oligonucleotide, has shown a dose-dependent effect on CSF t-tau concentrations but no beneficial effects on cognition.^9^

The rate of failures of large-scale clinical trials in Alzheimer’s disease suggests reconsidering the way we assess the biological efficacy of candidate anti-Alzheimer’s disease drugs. Indeed, Alzheimer’s disease is a multifactorial disorder in which several biochemical mediators interact at different levels. Similarly, in patients with Alzheimer’s disease, co-pathology could affect the clinical response to anti-amyloid antibody therapy.^10^ Despite this complexity, much of the current drug development is driven by a deterministic model of the disease that focuses on a single pathway.^11^ A missing piece in the Alzheimer’s disease puzzle concerns how misfolded proteins spread throughout the brain.^12^ Over the past 20 years, several studies have revealed that the brain is organized into a specific hierarchical architecture, referred to as the brain connectome. The architecture of the connectome seems linked with the pathophysiological spreading of misfolded proteins. Regions with strong connectivity tend to accumulate similar levels of misfolded proteins in early Alzheimer’s disease phases^13,14^ and in the presence of co-pathologies.^15^ Moreover, brain Aβ and tau accumulation are associated with breakdown of brain connectivity, which may in turn influence both cognition and neuropsychiatric symptoms. Network alterations have been reported also in cognitively normal individuals with subjective memory complaints and *APOE ε4* genotype, a risk gene of sporadic Alzheimer’s disease, regardless of the Aβ status.^16^

These data suggest that, on the one hand, brain connectivity influences Alzheimer’s disease pathological processes, including misfolded proteins’ spreading early in the pathophysiological cascade. On the other hand, connectivity is regulated in a complex way by the main players in the pathogenesis of Alzheimer’s disease (Aβ, tau, neuroinflammation and *APOE* genotype), and may provide a common biological substrate for the interactions of these markers. Further, given that the human connectome is highly subject specific,^17^ we propose a new framework suggesting that brain connectivity may represent a relevant readout for understanding the overall clinical potential of drug candidates for treating Alzheimer’s disease. The insights outlined in this review represent a proposal for integrating the current evaluation of new clinical candidates for the treatment of Alzheimer’s disease.

## The brain connectivity architecture

The human brain, with its intricacy comprising ∼100 billion neurons and an estimated 100 trillion synapses, represents a very complex organ. At the macro-scale level, we can explore *in vivo* this tangled network with several approaches, including MRI. Functional MRI (fMRI) represents a valid tool to explore this (functional) level of neural communication. This technique measures changes in blood oxygenation level-dependent (BOLD) signals, reflecting neural activity by tracking variations in regional blood flow (Box 1). By applying resting-state fMRI (rsfMRI), several functional networks have been identified, serving sensorimotor functions or individual cognitive functions extending from memory to attentional/executive functions. This architecture has been referred to as the functional connectome. These circuits exhibit specific topological features and a certain degree of involvement with behaviour in both health and disease,^18^ though the significance of resting activity in relation to behaviour remains a topic of ongoing discussion.^19^

Box 1 **Brain network connectivity**

- BOLD signal: When a determined brain region becomes active, there is an increased demand for oxygen, prompting a surge in blood flow to that area. The difference between baseline and activation can be captured by the fMRI signal, measuring the magnetic properties of oxygenated and deoxygenated haemoglobin. By tracking alterations in the BOLD signal over time, fMRI creates functional maps of the brain.
- Task fMRI: Task fMRI creates functional maps of the brain, contrasting brain signals during the execution of a specific task and baseline resting state. This approach allows for the identification of activated regions during specific tasks.
- rsfMRI: Using fMRI during resting-state conditions (that is, when individuals are not actively engaged in a task), it is possible to assess how the signal of brain regions at rest is synchronized, thus they are connected into resting-state neural networks, a measure referred to as FC. Although vascular in nature, rsfMRI is considered a reliable proxy of neural activity, showing that resting brain activity follows specific spatiotemporal trajectories organized into neural networks.
- dMRI: By applying gradient pulses in different directions, dMRI induces a phase shift in the MRI signal proportional to water diffusion rates. Modulating the strength and timing of these pulses sensitizes the signal to water molecule mobility, revealing tissue microstructure and offering insights into both normal and altered white matter tracts. The tractogram computed from dMRI data represents the structural connectome.

An intriguing aspect revealed by rsfMRI studies concerns the concept of divergent connectivity, wherein various neural networks exhibit anti-correlated patterns: when one network is active, another tends to be deactivated. A prime example of this phenomenon was observed in the default mode network (DMN) and the dorsal attention network (DAN). The former, associated with self-referential and memory processes, typically shows increased connectivity during rest and decreased connectivity during task engagement. Conversely, DAN, involved in attentional control and external task-oriented processing, exhibits the opposite pattern. This framework was enriched by the triple network model proposed by Menon^20^ by incorporating the salience network (SN) as a mediator between internal (DMN) and attentional networks, facilitating switches between network states.

Brain connectivity can also be assessed in terms of structural connections by means of diffusion MRI (dMRI). This technique leverages the Brownian motion of water molecules, influenced by tissue microenvironments (Box 1). Different techniques can be applied to dMRI, providing insights into specific properties of brain structural connectivity. Tractography maps the 3D pathways of nerve fibre bundles in the brain by tracing the movement of water molecules along white matter tracts. This technique is valuable for reconstructing brain tracts that contribute to the orchestration of both sensory and cognitive functions. This analysis complements the functional connectome. White matter tracts allow the transmission of neural signals between regions, constraining the patterns of correlated activity observed in rsfMRI data. Damage to specific tracts has an influence on the functional connectivity (FC) between distal regions connected through affected structural pathways.

**Figure 1:**
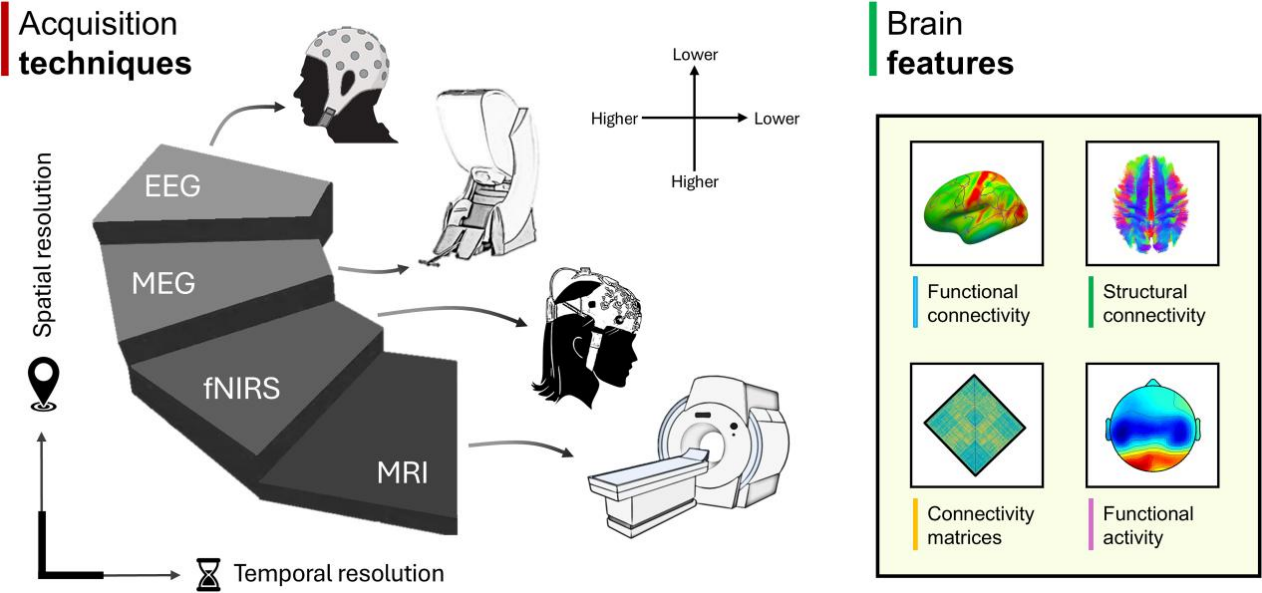
**Brain connectivity across multiple *in vivo* techniques.** Different *in vivo* techniques can be used to study brain connectivity, each offering different levels of spatial and temporal resolution. MRI provides the highest spatial resolution, whereas fNIRS, MEG and EEG offer superior temporal resolution, though at the expense of spatial precision. These techniques allow the extraction of a range of brain features, including functional and structural connectivity maps, connectivity matrices and functional activity across different frequency bands, providing insight into the relationships between pathophysiological mechanisms and brain communication. A conceptual representation of space and time is provided in the figure to highlight the higher (and similar) temporal resolution between EEG and MEG, compared with fNIRS and MRI. Similarly, the spatial resolution is represented arbitrarily with MRI exhibiting the finer spatial resolution.

It is important to note that other techniques can be used to assess brain FC, such as EEG, magnetoencephalography (MEG) and functional near-infrared spectroscopy (fNIRS). Several studies applied EEG or MEG in Alzheimer’s disease, revealing complex changes that occur in functional brain networks during early Alzheimer’s disease stages.^21,22^ While EEG and MEG excel in temporal resolution (milliseconds), capturing rapid neural dynamics, similarly to fNIRS (although not as high as that of EEG or MEG), their spatial resolutions are lower compared with MRI (Fig. 1). In contrast, rsfMRI and dMRI capture whole-brain connectivity with finer spatial resolution (up to 1 mm), which is essential for understanding the intricate architecture of functional networks in Alzheimer’s disease. Furthermore, rsfMRI and dMRI can be seamlessly integrated into the routine clinical MRI examinations already conducted for these patients. This integration has the potential to reduce both the time and cost of diagnosis, as no additional examinations are needed—only an extension of the prescheduled clinical examination. In the following sections, we will discuss how macro-scale connectivity mechanisms are altered in the Alzheimer’s disease continuum, providing insights on the inclusion of these measures in clinical trials.

## Brain connectivity in Alzheimer’s disease

An operational framework, called ATN from the biological markers of (A)myloid-β, (T)au and (N)eurodegeneration, has facilitated a transition from a clinical perspective to a biological viewpoint of Alzheimer’s disease, decoupling diagnosis from discrete clinical stages.^2,23^ Classification into ATN stages requires the designation of individuals as Aβ, tau and neurodegeneration positive or negative. The ATN system may be extended by adding specific brain fingerprints, such as connectivity outcomes from rsfMRI and dMRI. Indeed, the connectome may represent the biological substrate for the spreading of misfolded proteins, a model referred to as the molecular nexopathy model.^24^ In Alzheimer’s disease, the build-up of misfolded proteins initially localizes in brain regions characterized by heightened metabolism and FC.^25-27^ Subsequently, proteins spread to other regions via functional and structural connections. The cell-to-cell transfer of protein aggregates, akin to ‘prion-like’ behaviour, entails the release of proteins from one neuron to recipient neurons through synaptic contacts.^28-30^ This process is believed to be facilitated by the interconnectivity of neural networks in the brain, particularly through highly interconnected regions.^12^ In line with this network-molecular coupling, Alzheimer’s disease could be conceptualized as a network-based syndrome. The DMN, a set of functionally connected regions encompassing posterior cingulate, temporal, parietal and medial frontal cortices and linked with memory performance, has been demonstrated as being highly vulnerable in Alzheimer’s disease.^14^ In preclinical Alzheimer’s disease, Aβ begins to accumulate predominantly within the DMN and simultaneously affects brain connectivity.^14^ Studies in cognitively normal carriers of APP, PSEN1, or PSEN2 mutations or the *APOE ε4* allele, found that alterations of DMN occur prior to the occurrence of clinical symptoms.^31^ Notably, changes in FC are observed already in mutation-carrying children (PSEN1), suggesting that connectivity changes can occur decades prior to symptoms.^32^ To draw a comparison, functional network alterations could be analogous to what advanced diffusion microstructural imaging represents in the field of multiple sclerosis. While conventional imaging methods such as structural MRI may overlook changes extending beyond the visible lesion, advanced dMRI approaches have the capacity to reveal subtle alterations in both white and grey matter regions, indicating early disruptions.^33^ Consequently, even apparently normal white and grey matter exhibit alterations. Likewise, in Alzheimer’s disease, network alterations may reveal early pathological abnormalities that conventional MRI or molecular imaging techniques cannot detect. This prompts the assumption that network alterations could serve as a surrogate marker for underlying early Alzheimer’s disease molecular mechanisms.

Interestingly, connectivity alterations do not follow a linear pattern over the course of Alzheimer’s disease. The early Aβ accumulation (first) phase seems to be characterized by increased FC, which may subsequently hasten the spread of tau (second phase).^34^ Early brain functional alterations and neural hyperactivity seem to precede detectable amyloid plaques, suggesting they could alter or initiate the dynamics of proteinopathies. Subsequently, this activity-dependent modulation of tau release results in the progressive decline of FC with increased neocortical tau pathology.^35^ According to this model, tightly connected neural networks are marked by similar levels of tau accumulation, while high connectivity represents a load-shifting process transiently serving a potential compensatory role. Specifically, a time-dependent alteration of network balance is evident in the transition from hyper- to hypo-connectivity within and between networks. The former describes a connectivity state characterized by heightened integration among various brain regions compared with a normal physiological state. Conversely, hypo-connectivity refers to a less integrated connectivity pattern, typically observed between regions within the same network or among brain regions serving as hubs that are highly interconnected. Supporting this model, abnormal connectivity in mutation-carrier children was characterized by increased DMN FC.^32^ On the contrary, cognitively unimpaired carriers with autosomal dominant mutations showed reduced DMN functional patterns.^36^ Mutation carriers were on average 8 years from expected symptom onset and 13 years from expected dementia, suggesting a shift in brain connectivity abnormalities as the pathology progress. Similarly, autosomal dominant Alzheimer’s disease mutation carriers compared with non-carriers showed a positive correlation between blood levels of neurofilament light and DMN deterioration. This association explained significant variance in cognition. These studies support the *dual-stage* model whereby Alzheimer’s disease–related and gene-related pathological mechanisms induce alterations to local excitatory-inhibitory balance in the DMN, driving hyperexcitability leading to tau accumulation. Yet, the explanation for this divergent pattern remains unclear. While some research suggests that heightened FC may serve as a compensatory mechanism and following decreased connectivity reflect the harmful effects of Alzheimer’s disease progression, there is a proposition that increased connectivity might be associated with the early malfunctioning of specific neural pathways (e.g. GABA).^37^ Further studies should evaluate whether hyper-connectivity holds a truly compensatory or a detrimental effect. These temporally divergent FC patterns have also been spatially assessed within different brain networks. In the symptomatic stage of Alzheimer’s disease, DMN hypo-connectivity seems accompanied by hyper-connectivity of anti-correlated networks, such as the SN.^38^ According to the triple network theory,^20^ SN acts as a gate suppressing DMN activity in response to a specific stimulus/task. SN hyper-connectivity could reflect unbalanced network properties, preventing DMN connections and thus resulting in suppressed DMN FC patterns. Independent studies reported similar divergent within-network FC patterns, mainly mapping to the SN and the DMN. Overall, increased SN connectivity could be linked with detrimental clinical symptoms.^39^ Notably, intervention aimed at modulating SN connectivity in Alzheimer’s disease by means of non-invasive brain stimulation (NIBS) led to improved clinical symptoms, suggesting a causal link between SN connectivity patterns and disease severity.^40^

Alzheimer’s disease exhibits substantial inter-individual variability in both clinical symptoms and progression. This variability can challenge the use of brain connectivity as a predictive tool. However, recent research suggests that individualized brain connectivity patterns, known as ‘connectome fingerprints’, may help account for this heterogeneity by providing stable, subject-specific signatures of brain network architecture.^41^ These fingerprints offer potential for more personalized diagnostic and treatment approaches. Moreover, these findings emphasize the importance of focusing on individual variability rather than group differences in Alzheimer’s disease studies. Further, brain connectivity showed specificity in terms of clinical phenotypes. Patients with Alzheimer’s disease may present with an atypical profile at clinical onset, involving impairment primarily in behavioural/executive functions,^42,43^ language domain,^44,45^ or visuospatial abilities.^46^ These distinct clinical phenotypes correspond to unique network connectivity patterns, mapping to networks that support executive/attentional, language, or visuospatial functions, in accordance with the clinical presentation.^47^ Additionally, the dynamic nature of brain connectivity in Alzheimer’s disease, shifting from hyper- to hypo-connectivity as the disease progresses, provides further insights into the mechanisms underlying individual variability in Alzheimer’s disease symptoms and progression. Yet, other factors may influence the association between connectivity and symptom/progression at both the individual and phenotype levels. Future studies should focus on integrating connectivity data with genetic, molecular and clinical markers to improve predictions of disease progression and treatment response, allowing for tailored therapeutic strategies.

While FC represents the most studied connectivity features in Alzheimer’s disease, structural connectivity is assuming increasing relevance. Recently, it has been demonstrated that it is possible to predict molecular accumulation using machine learning algorithms constrained by diffusion-derived structural connections.^48^ These models can predict the concentration of molecular pathology in a specific brain region at a given time point, leveraging the concentration of pathology in connected regions at an earlier time point. On the other hand, proteinopathies could affect structural connections and white matter integrity, which deserve further investigation. Overall, functional and diffusion studies confirm the hypothesis that brain connectivity could provide the biological substrate for molecular propagation both at synaptic and axonal levels and could in turn be affected by such pathological mechanisms. Notably, this concept can be applied to pathological mechanisms other than proteinopathies, such as microglial activation and neuroinflammation.^49,50^

**Figure 2:**
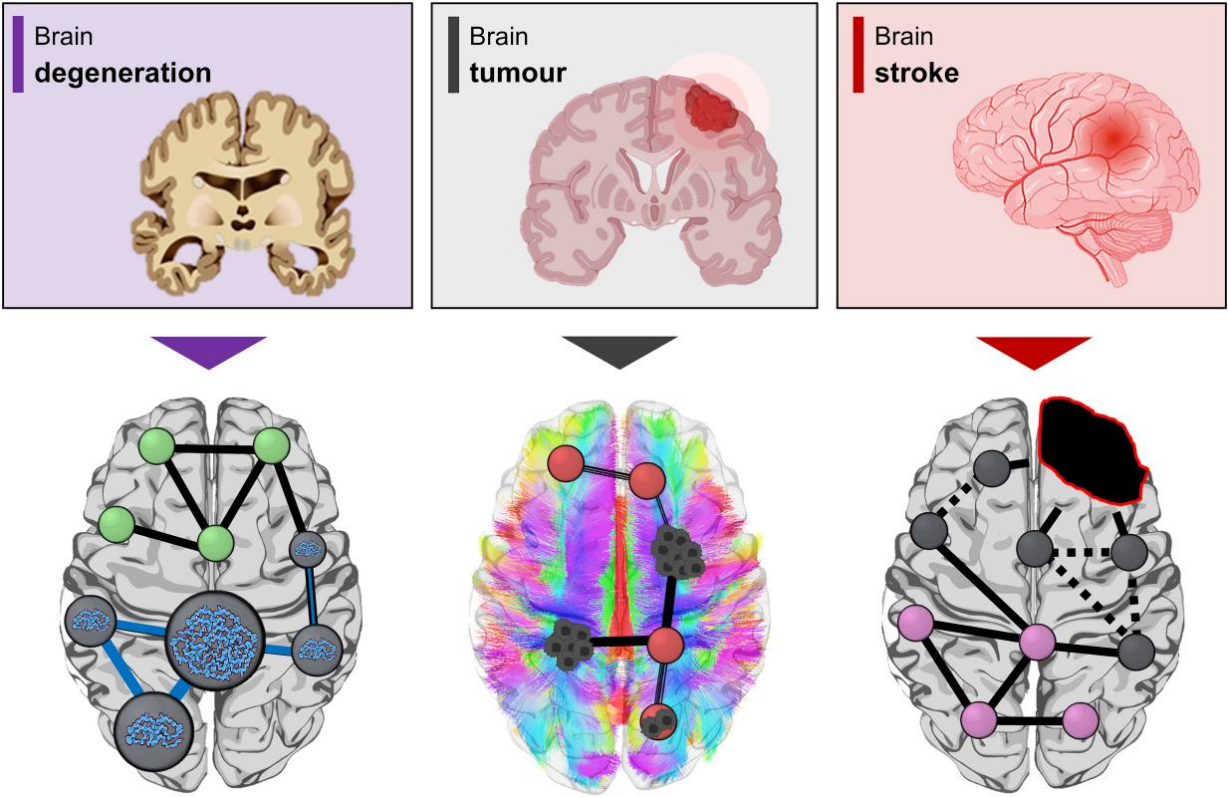
**Brain connectivity across different neurological diseases.** The brain connectome (both functional and structural) may represent a common substrate linking pathophysiological mechanisms to the clinical phenotypes of neurodegenerative diseases, gliomas and stroke. In neurodegenerative diseases, the connectome could serve as the biological substrate for the spread of misfolded proteins. In gliomas, tumour cells may spread along axonal pathways, suggesting that tumours growing in regions of high connectivity are more likely to recur, thus worsening prognosis. In stroke, connectivity diaschisis mechanisms have been described, indicating that the degree of disability after a stroke is more closely related to the lesion’s impact on brain connectivity than to the size of the lesion.

Furthermore, brain connectivity could represent a common substrate for several neurological diseases. As discussed, in Alzheimer’s disease, misfolded proteins can spread along specific connectivity pathways.^12,51^ This relationship has also been observed in other proteinopathies, such as Huntington’s disease^52^ and Parkinson’s disease.^53^ Interestingly, studies on brain diseases associated with completely different mechanisms, such as stroke or glioblastoma, suggest a ubiquitous role of brain connectivity as an interface between pathophysiological mechanisms and clinical phenotypes (Fig. 2). In stroke, a phenomenon known as functional diaschisis has been described, highlighting how the extent of network disconnectivity from a specific lesion can predict both acute disability and the degree of recovery.^54,55^ Recent studies have also emphasized the significant role of brain connectivity in the prognosis of glioblastoma, the most malignant brain tumour. Specifically, brain network connectivity may help predict overall survival in these patients^56^ and offer insights for the development of novel, effective interventions, a critical unmet need in this field.^57^ Overall, these studies suggest that brain connectivity may serve as a key factor in understanding the mechanisms underlying a variety of neurological conditions and could provide a valuable target for therapeutic interventions.

## Anti-Alzheimer’s disease drugs effects on brain connectivity: a novel integrative framework

FC represents a potentially useful candidate biomarker in Alzheimer’s disease animal models, as it helps to detect alterations related to the disease progression, by identifying differences in specific networks even at the early disease stages.^58^ Connectivity alterations, associated with progressive grey matter microstructural integrity alterations,^59^ as well as reduced DMN connectivity^58^ were reported in TgF344-AD compared with wild-type rats. Hippocampal connectivity was shown to decrease in the triple transgenic Alzheimer’s disease mouse model (3×Tg-AD) before Aβ plaques overaccumulation, when related to wild-type controls. Such early-stage alterations in hippocampal connectivity represent potential translational biomarkers to humans in Alzheimer’s disease preclinical stages.^60^ FC breakdown was also found in transgenic mouse models of tauopathy relative to their wild-type littermates. Interestingly, both pro-aggregant and anti-aggregant transgenic mice exhibited almost equivalent connectivity strengths across all networks when the transgene human tau repeat domain was fully expressed. Hence, it was reported a substantial decrease of FC induced by increased soluble tau, independent of its intrinsic predisposition to aggregation. Thus, as the increased amount of soluble tau can lead to toxic effects, it represents a potential early-stage drug target using functional networks analysis.

At present, studies conducted on Alzheimer’s disease animal models to explore the potential effects of drugs on brain connectivity are restricted. In APP23×PS45 transgenic mice, neuronal hyperactivation due to Aβ accumulation was initiated by the suppression of glutamate reuptake, using DL-threo-β-benzyloxyaspartic acid and occurred only in neurons with pre-existing baseline activity, thus fuelling a vicious cycle.^61^ These findings support a model in which Aβ-dependent neuronal dysfunction can be activated before plaque formation. Other studies conducted in PDAPP and Tg2576 transgenic mice demonstrated that passive immunotherapy with Aβ-targeting antibodies (3D6 and β1) worsened abnormal neuronal hyperactivity and cognitive deficits.^62^ On the other hand, treatment with the BACE1 inhibitor NB-360 normalized abnormal neuronal activity and attenuated memory deficit in APP23×PS45 transgenic mice.^63^ These positive results in mouse models of Alzheimer’s disease were not replicated by other BACE1 inhibitors such as verubecestat, lanabecestat, elenbecestat, atabecestat and umibecestat.^64^ Indeed, the cognitive worsening observed in clinical trials with these BACE1 inhibitors to date, suggests that normalization of neuronal activity may not be achieved in humans. Further, rsfMRI and other imaging modalities are potentially useful to determine possible deleterious effects of drug-induced reduction of brain Aβ burden on neuronal activity and FC in networks implicated in cognition.

To date, few studies have explored the effects of anti-Alzheimer’s disease drugs on brain connectivity in patients with Alzheimer’s disease. Some of these studies demonstrated increased functional activation and connectivity, as well as positive responses of critical brain metabolites reflecting neuronal status and functionality.^65^ Regarding symptomatic treatments, cholinesterase inhibitors and memantine increased both activation and FC in multiple brain regions and networks; brain functional responses were often correlated with cognition. In a pivotal study, Lorenzi *et al*.^66^ demonstrated increased resting DMN activity in the precuneus of patients with Alzheimer’s disease 6 months after starting the treatment. A more recent study evaluated cognitive performance in patients with mild Alzheimer’s disease who were receiving cholinesterase inhibitors (donepezil 10 mg, rivastigmine 12 mg or galantamine 24 mg).^67^ Following patients’ assessments at 10–12 month intervals, drug ‘responders’ had significantly higher mean FC of the right hippocampus at baseline than ‘non-responders’. This model achieved an accuracy of 78% suggesting that FC may help to predict response to treatment.^67^ At baseline, the two groups did not differ in terms of demographic, cognitive function, CSF Alzheimer’s disease biomarkers (Aβ_1–42_, p-tau181, t-tau), or other regional brain FC.^67^ A longitudinal rsfMRI analysis was carried out to detect functional network topological changes in nine patients with mild-to-moderate Alzheimer’s disease, before and after treatment with oral rivastigmine for 24 weeks at growing doses (1.5 mg twice a day to 12 mg/day)—at both systemic and regional levels—versus nine healthy controls. Patients with Alzheimer’s disease showed post-treatment improvement in both cognitive and Activity of Daily Living Scale scores, while imaging analysis did not show marked post-treatment changes in the systemic network measures. However, authors reported a regional network (bilateral caudate and right superior temporal pole) post-treatment FC increase in these patients.^68^ Similar regional connectivity effects were reported following donepezil treatment in patients with Alzheimer’s disease treated for 3–6 months.^69-71^

Recently, a review of EEG studies examined the effects of various acetylcholinesterase inhibitors (i.e. galantamine, rivastigmine, donepezil, tacrine and physostigmine) on the brain activity of patients with Alzheimer’s disease through EEG analysis. The findings indicated that these drugs can significantly influence the brain waves of patients with Alzheimer’s disease. However, the effects on the alpha band were inconsistent, showing both decreased and increased patterns after medication intake.^72^ These results are particularly relevant in the context of a recent study that demonstrated a synergistic relationship between early Aβ deposition and neurophysiological enhancement of high-frequency brain activity, evidenced by increased alpha-band and decreased delta-band activity.^73^ Early tau accumulation in the temporal cortex may interact with Aβ to drive additional pathological processes. This interaction is thought to result in a transition from increased oscillatory activity during the preclinical phase of the disease to a widespread slowing of brain activity as the disease progresses into the prodromal and clinical Alzheimer’s disease stages.^73^

Finally, it is worth mentioning the results recently obtained with NIBS in patients with early Alzheimer’s disease with biomarker-proven Aβ pathology.^74^ After 20 sessions of personalized NIBS or sham stimulation targeting the parietal area, active treatment showed a higher mean change in both cognitive and clinical outcomes compared with the sham. Further, increased FC within DMN regions was associated with cognitive improvements. This pilot randomized study indicated that the positive effects of NIBS on cognitive performance when targeting DMN regions in early Alzheimer’s disease were driven by plastic changes in the hippocampal-DMN network.^74^ Similar results were obtained in patients with mild Alzheimer’s disease targeting DMN or SN hubs, suggesting a differential effect on clinical and cognitive variables.^40^ These results suggest that targeting network connectivity could represent a valid choice aimed at maximizing the clinical/cognitive benefits of brain stimulation therapies.

**Figure 3:**
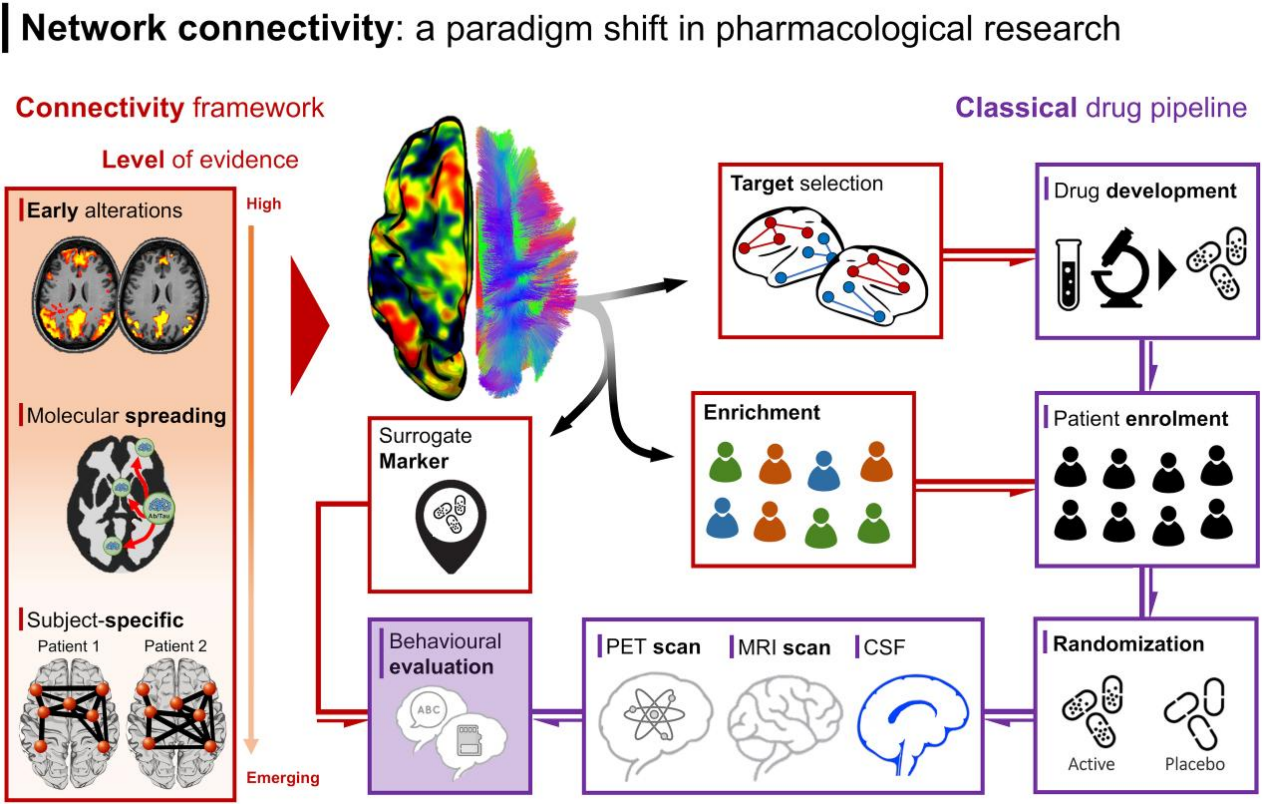
**Including brain network information in the pharmacological field.** Left panel: Functional and diffusion brain imaging can be used to assess brain network connectivity by examining time-dependent fluctuations in neural signalling and water molecule displacements, respectively. Different levels of evidence indicate network failure in Alzheimer’s disease patients: a growing body of literature suggests brain network alterations in Alzheimer’s disease can be observed early in the pathophysiological cascade; recent studies consistently reported that connectivity failure could promote or hasten the pathophysiological processes; emerging literature suggests that connectivity can be used as a fingerprint to assess subject-specific changes. Right panel: Brain connectivity assessed with either functional or diffusion imaging could enter the design of clinical trials at different stages: (i) it could enrich clinical trials by contributing to the selection of patients at a specific stage of the Alzheimer’s disease pathophysiological cascade; (ii) it could aid in the selection of specific brain regions exhibiting connectivity abnormalities but normal-apparent pathological accumulation for therapeutic target selection and (iii) it could serve as a surrogate marker of efficacy, assessing whether the intervention is able to normalize aberrant connectivity in treated patients.

Overall, the available literature suggests that symptomatic treatments can modify regional network properties, highlighting the potential of using FC measures as biomarkers for monitoring the efficacy of these Alzheimer’s disease drugs. Similarly, this complex picture suggests the potentiality for a paradigm shift in the development of novel clinical trials in the context of anti-Aβ and anti-tau monoclonal antibodies. Based on these premises, we suggest that a more holistic approach, incorporating brain connectivity measures, may provide a clearer assessment of drug efficacy, with a threefold benefit (Fig. 3).

First, this framework could enhance our understanding of the relationship between molecular accumulation and spreading mechanisms, thereby informing new potential targets. Specifically, the connectome could help in the development of novel treatments by considering the impact of Alzheimer’s disease–related pathology on network connectivity,^34,75^ or tailoring interventions based on the unique connectome fingerprint of individuals, such as in the field of NIBS,^76^ or used in conjunction with digital twins models. For instance, brain connectivity, when modelled within the Virtual Brain framework,^77^ offers a powerful approach to understanding the dynamic interactions within neural networks and their alterations in neurological and psychiatric disorders. This computational framework simulates brain activity and connectivity, enabling the modelling of neural networks and the prediction of how brain dynamics are affected by various factors. Ultimately, it facilitates the creation of digital brain twins—personalized brain models for patients. This framework shows great promise in identifying biomarkers and predicting how different interventions, such as pharmaceutical agents, impact brain function and connectivity in real time. For instance, in pharmacoresistent epilepsy, virtual models of patients’ brains could guide more effective treatments by pinpointing the exact zones responsible for seizures, aiding neurosurgeons in planning interventions. In Alzheimer’s disease, this approach has been used to simulate the effect of introducing the NMDA receptor antagonist memantine, examining its impact on brain connectivity signals.^78^ The results provided a potential mechanistic explanation for memantine’s effectiveness and serve as a general testbed for evaluating the efficacy of various treatment strategies.

Second, this integration could serve as a strategy for enriching patient selection in clinical trials based on connectivity profiles and potential underlying comorbidities. As for the inclusion of the hippocampus volume, qualified by the EMA for the purpose of enrichment in Alzheimer’s disease clinical trials at the predementia stage (CHMP, EMA/CHMP/SAWP/809208/2011), assessments of network alterations could be used as an enrichment strategy or for predicting which patients are likely to benefit from specific treatments based on their connectome alterations. Further, brain connectivity could provide insights not only into the staging of different patients based on non-linear alterations (hyper- versus hypo-connectivity), but also into whether cognitive deficits are more likely associated with the primary pathophysiology or comorbid conditions,^79,80^ thus aiding in patient enrolment.

Finally, brain connectivity could act as a surrogate marker of efficacy, allowing for a more dynamic and network-centred evaluation of drug performance. Connectivity could represent an effective measure not only for evaluating clinical efficacy but also as a surrogate marker of efficacy in interim analyses of studies with adaptive design. This could be particularly relevant in the context of clinical trials assessing off-labels drugs or testing whether failure in previous trials could be interpreted on the light of connectivity. Additional positive examples of using connectivity as a biomarker in drug discovery can be found across different brain disorders. For instance, in Parkinson’s disease, changes in FC within motor networks have been used to assess the efficacy of dopaminergic treatments.^81^ Similarly, in major depressive disorder, antidepressants have been shown to normalize connectivity patterns, with these changes correlating with clinical improvements.

These examples underscore the potential of connectivity-based biomarkers to serve as early indicators of treatment response. Applying this approach to Alzheimer’s disease could pave the way for new interventions and provide a means to reassess previously tested drugs that have shown limited or no benefit.

## Conclusion

The brain is a complex system organized into networks. The connectome framework allows a shift from a localizationist perspective, where brain regions are individually and separately conceptualized and analysed, to a system-level viewpoint that considers the brain as a network of interacting components. A breakdown of this system could predict cognitive clinical symptoms observed in brain diseases. The bidirectional interplay between molecular mechanisms and neural connectivity suggests that Alzheimer’s disease–related pathophysiological processes actively interact with the brain connectome. While many conclusions regarding connectivity changes are based on studies conducted in animal models of Alzheimer’s disease, which may not fully reflect the complexity of the human disease, these models provide crucial insights into early-stage brain network alterations. For example, hippocampal connectivity has been shown to decrease in animal models, such as the 3×Tg-AD mice, even before Aβ accumulation becomes detectable. These findings suggest that connectivity alterations could serve as early biomarkers, though their translational potential to humans remains to be fully validated in larger clinical studies. Recent evidence from preclinical and at-risk human populations, such as *APOE ε4 carriers*, supports the relevance of these observations, but larger, well-controlled trials are necessary to confirm whether connectivity changes can serve as reliable early markers or treatment indicators in Alzheimer’s disease.

So far, from a pharmacological point of view, the Alzheimer’s disease brain has largely been considered and treated in a localizationist manner, relying mainly on Aβ or tau biomarkers. The 2023 Alzheimer’s drug development pipeline^82^ reports 187 trials assessing 141 unique treatments. Among trials in Phase III (*n* = 55), the majority include measures of amyloid and/or tau accumulation as outcomes, while a small percentage (around 16%) include conventional structural MRI outcomes (e.g. volume or cortical thickness) and one study includes cerebral blood flow assessed with arterial spin labelling neuroimaging. None of these trials included connectivity measures as outcomes. We must learn from previous failures and limited clinical results of anti-Aβ and anti-tau drugs, shifting the focus to new frameworks, including models that take into account the complex and interconnected architecture of the brain. Connectivity-derived measures should be integrated into clinical trial designs, potentially revolutionizing pharmacological approaches. New drug candidates need to be evaluated during early stages of development for their global effects on brain connectivity to better predict patients’ cognitive and clinical performance, which is indeed a result of overall brain functioning. In conclusion, while brain connectivity measures are not yet widely adopted as primary endpoints in clinical trials for Alzheimer’s disease, preliminary studies have shown that FC changes often precede Aβ and tau pathology and may provide an earlier indication of disease progression. Connectivity-based assessments, when integrated with traditional biomarkers, could offer a more holistic evaluation of drug efficacy. As the field evolves, incorporating brain connectivity as a secondary endpoint in adaptive trial designs could better capture the complex, network-based dysfunction of Alzheimer’s disease and potentially serve as surrogate markers for early treatment response. Such integration would provide a more comprehensive framework for evaluating disease-modifying drugs in future clinical trials.

## Data Availability

Data sharing is not applicable to this article as no new data were created or analysed in this study.
